# Long-Term Outcomes of Valve Replacement With Mechanical Prosthesis in Patients With Valvular Heart Disease: A Single-Center Retrospective Study

**DOI:** 10.7759/cureus.84655

**Published:** 2025-05-22

**Authors:** Devendra Saksena, Ankita Choudhary, Sandeep Varma, Shivprasad Shetty, Vaibhav Jain

**Affiliations:** 1 Cardiovascular and Thoracic Surgery, Bombay Hospital and Medical Research Centre, Mumbai, IND; 2 Cardiology, Bombay Hospital and Medical Research Centre, Mumbai, IND

**Keywords:** major adverse cardiovascular events, mechanical valve, rheumatic heart disease, survival rate, valvular heart disease

## Abstract

Background

Significant valve disease requires surgical intervention, either valve repair or valve replacement. For minor disease, balloon dilation is a possibility. The choice between mechanical and bioprosthetic valves requires a judgment regarding the benefits and risks of each procedure. A mechanical prosthetic valve requires lifelong anticoagulation, whereas a bioprosthetic valve tends to degenerate over a few years, with faster degeneration observed in younger patients.

Objective

To assess the survival outcomes, postoperative complications, and reoperation rates in patients who underwent prosthetic mechanical valve replacement with acenocoumarol and low-dose aspirin (75 mg), with adequate International Normalized Ratio (INR) monitoring.

Methods and materials

This was a retrospective study involving data from patients who underwent mechanical cardiac valve replacement between 1971 and 2022. This study adhered to the principles outlined in the Declaration of Helsinki and received approval from the institutional ethics review board of Bombay Hospital (Regn. No: ECR/296/Inst/MH/2013; Date: 08/12/2021).

Results

A total of 768 patients were included. The mean overall survival rate was 35.2%, and it was higher in men than in women. The majority of patients belonged to a younger age group (≤18 years: 6.3%, 19-40 years: 47.7%, 41-60 years: 42.2%, >60 years: 3.9%). The mean overall survival rate was higher in men (37.4%) than in women (28.4%). In the first year post-surgery, females experienced Major Adverse Cardiac and Cerebrovascular Events (MACCE) at a rate of 11.1 person-years, while males had none. Among patients classified as New York Heart Association (NYHA) class III, the incidence rate of MACCE was 2.7 person-years, whereas for NYHA class IV patients, it was 8.3 person-years. These trends persisted to some extent at the fifth year post-surgery.

Conclusion

Survival outcomes were influenced by factors such as age, sex, type of valve replacement, and NYHA class, with certain subgroups showing better survival rates. The first year post-surgery presented a higher incidence of MACCE, which declined over time. Mechanical valve replacement with appropriate anticoagulation can offer favorable long-term outcomes, particularly in younger patients. However, early postoperative risks, especially in women and those with advanced heart failure, highlight the need for individualized care and close monitoring. Future research should aim to refine patient selection, explore sex-based outcome disparities, and optimize anticoagulation strategies to further improve survival and quality of life in this population.

## Introduction

Rheumatic heart disease (RHD) is a common and serious complication that arises from single or frequent attacks of rheumatic fever, resulting in decreased mobility, fibrosis, and calcification of the valve cusps and sub-valvar apparatus. It accounts for nearly 250,000 deaths worldwide each year, especially among younger age groups, affecting them in their prime of life [1,2]. Over a span of 20-30 years, RHD can culminate in valvular stenosis and/or regurgitation, affecting the mitral valves (50-60%), both the aortic and mitral valves (20%), the tricuspid valves (10%), and rarely, the pulmonary valves. The core management strategy for valvular disease involves surgical intervention, which can include valve replacement or repair [2]. Surgical valve replacement or repair remains the gold standard in the treatment of valvular heart disease (VHD) [3].

The current prosthetic valves employed for replacing diseased aortic and mitral valves are mainly of two types: mechanical and bioprosthetic. Mechanical prosthetic heart valves (MPVs) offer easy availability, comparatively lower costs, and long-term durability; however, they necessitate lifelong anticoagulation [4,5]. Bioprosthetic heart valves (BPVs) do not require lifelong anticoagulant therapy and have excellent hemodynamic properties; however, BPVs are associated with early structural valve degeneration [6-8]. Guidelines from the American Heart Association (AHA)/American College of Cardiology (ACC) and European guidelines also recommend mechanical valves in VHD patients aged <50 years [8,9]. Bioprosthetic valves have relatively slower degeneration in elderly patients. There is a great need in developing countries to develop a valve with durability exceeding 30-50 years. This highlights the importance of studying long-term event-free survival rates following prosthetic valve surgery in developing countries.

Anticoagulation therapy is absolutely necessary in patients with prosthetic valves [10,11]. An increased risk of bleeding and thromboembolism (stroke and valve thrombosis) has been observed in patients with poor compliance to anticoagulation therapy and failure to maintain international normalized ratio (INR) within the recommended range (2.5 to 3.5). This study has followed the anticoagulation guidelines suggested by Saksena D et al. [6,12].

Some patients undergoing valve replacement require redo surgery, mainly due to bioprosthetic valve degeneration, and occasionally due to infective endocarditis or paravalvular leak. In mechanical valves, pannus development can sometimes necessitate redo surgery. While redo surgery has become safer, it still poses a considerable burden, socially, financially, and occasionally in terms of morbidity. Some long-term studies are available for mechanical valves; however, in India, there are no major studies documenting long-term survival beyond three to four decades. We have attempted to study the long-term survival of patients for 30 years or more.

This study was conducted involving patients treated by a single surgeon at a single medical facility, spanning over fifty years of follow-up. In adolescents and young adults, the aim was to implant a prosthetic valve capable of lasting 30 to 40 years to avoid redo surgery. This study is unique in several aspects, e.g., single-center involvement, with patients operated on by the same surgeon and personally followed up by the operating surgeon over a period of four to five decades. To our knowledge, no similar study of this type exists. In addition, the insights gained from this study can aid in formulating guidelines for INR management, determining factors that influence the choice between a MPV and a BPV, and addressing other related concerns.

In light of the aforementioned literature, the present study aims to assess the survival outcomes, postoperative complications, and reoperation rates in patients who underwent prosthetic mechanical valve replacement with acenocoumarol and low-dose aspirin (75 mg), with adequate INR monitoring.

## Materials and methods

Study design

This was a retrospective study conducted at Bombay Hospital and Medical Research Centre, Mumbai, involving data from patients who underwent mechanical cardiac valve replacement between 1971 and 2022.

Ethical considerations

This study adhered to the principles outlined in the Declaration of Helsinki and received approval from the Institutional Ethics Review Board of Bombay Hospital (Regn. No: ECR/296/Inst/MH/2013; Date: 08/12/2021). Informed consent was not obtained due to the retrospective nature of the study and the use of anonymized clinical data for analysis.

Data collection

A total of 931 patient records were retrieved. After excluding cases with unavailable data, follow-up information for 768 patients was retrospectively analyzed. Demographic details, valve prosthesis type, valvular replacement, survival data, and postoperative complications were extracted from each patient record.

Patient selection

Patients who underwent mechanical cardiac valve replacement, with no prior history of stroke and managed with acenocoumarol and low-dose aspirin (titrated based on INR monitoring), were included in this study. Patients who underwent cardiac surgeries other than valve replacement were excluded.

Endpoints

The primary endpoint of the study was to assess the overall survival of patients who underwent prosthetic valve replacement and were managed with INR-based dose titration of acenocoumarol.

The secondary endpoint was to evaluate the incidence of major adverse cardiovascular and cerebrovascular events (MACCE), such as cerebrovascular events, myocardial infarction, major bleeding episodes, stroke, and cardiac revascularization. Additionally, the study aimed to assess the incidence of redo surgeries during the follow-up period and to determine the association between INR and time to therapeutic range (TTR).

Statistical analysis

Continuous data were presented as mean and SD, or median and IQR, as applicable. Categorical data were expressed as counts and percentages. Kaplan-Meier survival curves were constructed for the entire study population and various subgroups of interest to illustrate the primary endpoint. The log-rank test was used to assess differences between survival curves for the various subgroups. A p-value of less than 0.05 was considered to indicate statistical significance. Statistical analyses were performed using the SPSS version 24.0 (Chicago, IL).

## Results

Baseline characteristics of the patient

A total of 768 patients were included in this study. The proportion of male patients (63.0%) was higher than that of female patients (37.0%). Similarly, the majority of patients belonged to a younger age group (19-40 years: 47.7% vs. >60 years: 3.9%). The mean (SD) age of the patients was 38.7 years. The majority of patients had undergone mitral valve replacement (53.3%), followed by aortic (30.9%) and combined mitral + aortic valve replacement (15.4%). Most patients had a NYHA class IV presentation (64.3%), followed by class III (34.4%). Monoleaflet mechanical valves were used in the majority of patients (74.6%), followed by bileaflet mechanical valves (24.0%). The most commonly used mechanical valves were Medtronic Hall (70.7%), followed by St. Jude (13.9%), and ATS metallic (6.5%). All patients with atrial fibrillation underwent a modified Maze procedure with left atrial appendage ligation. The risk of redo surgery was observed in 13.7% of patients. The mean follow-up period was 10.8 years. Around 4.6% of patients underwent aortic root enlargement with aortic valve surgery, operated by the valve-in-valve procedure (Table 1).

**Table 1:** Demographic characteristics of patients. *Data not available for 379 patients; †Data not available for 472 patients.

Characteristics	Mean ± SD, (IQR) / n (%)
Age (in years)	38.7 ± 13.02, (3-71)
≤18	48 (6.3)
19-40	366 (47.7)
41-60	324 (42.2)
>60	30 (3.9)
Sex	
Male	484 (63.0)
Female	284 (37.0)
Rhythm	
Normal sinus rhythm (NSR)	616 (80.2)
Atrial fibrillation	152 (19.8)
Types of valve replacement	
Mitral	409 (53.3)
Aortic	237 (30.9)
Pulmonic	1 (0.1)
Mitral + Aortic	118 (15.4)
Mitral + Tricuspid	2 (0.3)
Mitral + Aortic + Tricuspid	1 (0.1)
NYHA classification	
III	264 (34.4)
IV	494 (64.3)
IV-c	10 (1.3)
Type of valve prosthesis	
Monoleaflet mechanical valve	664 (74.6)
Bileaflet mechanical valve	214 (24.0)
Caged ball valve	12 (1.3)
Types of prosthetic valves used	
Medtronic Hall	543 (70.7)
St. Jude	107 (13.9)
ATS metallic	50 (6.5)
Bjork–Shiley	26 (3.4)
On-X bileaflet mechanical valve	20 (2.6)
Starr-Edwards	12 (1.5)
Carbomedics	6 (0.7)
Lillehei-Kaster	2 (0.2)
Bicarbon valve	1 (0.1)
Omni Carbon	1 (0.1)
Left ventricular ejection fraction (LVEF)†	
Hyperdynamic	1 (0.3)
Normal	214 (72.3)
Mild dysfunction	37 (12.5)
Moderate dysfunction	19 (6.4)
Severe dysfunction	25 (8.4)
Aortic regurgitation	
No	412 (53.6)
Mild	168 (21.9)
Moderate	74 (9.6)
Severe	112 (14.6)
Calcific	2 (0.3)
Atrial fibrillation*	
Pre-operative atrial fibrillation	150 (19.5)
Post-operative atrial fibrillation	239 (31.1)
Pannus excision	
Yes	10 (1.3)
No	758 (98.7)
Patients who underwent aortic root enlargement with aortic valve surgery	
Yes	35 (4.6)
No	749 (95.4)
Redo surgery risk	
Yes	105 (13.7)
No	663 (86.3)
Follow-up	
Mean follow-up in years (SD)	10.82 (8.65)
Median follow-up in years (IQR)	9.56 (3.15-16.36)

Comparison of survival rates

The mean overall survival rate was 35.2%, higher in men (37.4%) than in women (28.4%). The median survival rate was 43.1%, significantly higher in men (43.1%) compared to women (34.2%), p=0.004 (Table 2).

**Table 2:** Comparison of survival rates based on Kaplan-Meier estimates and log-rank statistics. Estimation is limited to the largest survival time if it is censored. The p-value is based on the log-rank test (Mantel-Cox).

Sex	Mean^a^				Median				Chi-square	P-value
	Estimate	Std. Error	95% CI (Lower)	95% CI (Upper)	Estimate	Std. Error	95% CI (Lower)	95% CI (Upper)		
Female	28.4	1.358	25.78	31.09	34.2	5.101	24.18	44.17	8.86	P < 0.05
Male	37.4	1.393	34.67	40.13	43.1	8.288	26.85	59.34		
Overall	35.2	1.299	32.66	37.75	43.1	6.461	30.43	55.76		

Incidence and rates of MACCE stratified by sex, NYHA class, and type of valve replacement

The incidence and rates of MACCE varied based on sex, NYHA class, and type of valve replacement. In the first year post-surgery, females experienced MACCE at a rate of 11.1 person-years, while males had none. Among patients classified as NYHA class III, the incidence rate was 2.7 person-years, whereas for NYHA class IV patients, it was 8.3 person-years. Regarding valve replacement type, patients receiving a single valve had an incidence rate of 8.3 person-years, while for double valve recipients, it was 2.7 person-years. These trends persisted to some extent at the fifth year post-surgery, with variations observed across different patient groups (Table 3).

**Table 3:** Incidence and rates of MACCE stratified by sex, NYHA class, and type of valve replacement. MACCE: Major Adverse Cardiac and Cerebrovascular Events; NYHA: New York Heart Association.

Characteristics	1st Year n (person-years)	5th Year n (person-years)	Overall n (person-years)
Sex			
Female	4 (11.17)	6 (1.35)	37 (0.0012)
Male	0 (0.00)	8 (1.79)	41 (0.0014)
NYHA Classification			
Class III	1 (2.79)	4 (0.90)	29 (0.0010)
Class IV	3 (8.38)	10 (2.24)	49 (0.0016)
Type of Valve Replacement			
Single valve	3 (8.38)	13 (2.92)	66 (0.0022)
Dual valve	1 (2.79)	1 (0.22)	12 (0.0004)
Total	4 (11.17)	14 (3.14)	78 (0.0026)

Mean dose of acenocoumarol and INR levels

Throughout the follow-up period, the mean INR levels and acenocoumarol doses varied. In the initial three months, the mean INR was 2.64, with patients receiving a mean dose of 2.34 mg/day of acenocoumarol. Over the next few months, slight fluctuations were observed in both mean INR and acenocoumarol dose, with the highest mean INR observed in the >180.1 months follow-up group (2.64) and the highest mean dose of acenocoumarol noted in the 120.2-180.1 months follow-up group (2.77 mg/day). Despite these variations, mean INR levels generally remained within the therapeutic range of 2.0 to 3.5, ensuring appropriate anticoagulation management throughout the study period. These findings highlight the importance of INR monitoring and dose adjustments to maintain therapeutic anticoagulation levels and mitigate the risk of complications in patients undergoing valve replacement surgery (Table 4).

**Table 4:** Mean dose of acenocoumarol and INR levels. INR: International Normalized Ratio.

Follow-up (months)	N	INR, Mean (SD)	Acenocoumarol Dose (mg/day), Mean (SD)
≤3	219	2.64 (1.32)	2.34 (0.99)
3.1-6.0	206	2.44 (1.16)	2.35 (0.98)
6.1-9.0	183	2.31 (0.84)	2.48 (0.94)
9.1-12.0	168	2.51 (1.04)	2.46 (1.07)
12.1-36.0	348	2.37 (0.70)	2.63 (0.99)
36.1-60.0	313	2.51 (0.79)	2.75 (1.04)
60.1-120.1	370	2.47 (0.78)	2.71 (1.07)
120.2-180.1	294	2.59 (0.87)	2.77 (1.20)
>180.1	231	2.64 (0.87)	2.64 (1.13)

Gender specificity and survival outcome

**Figure 1:**
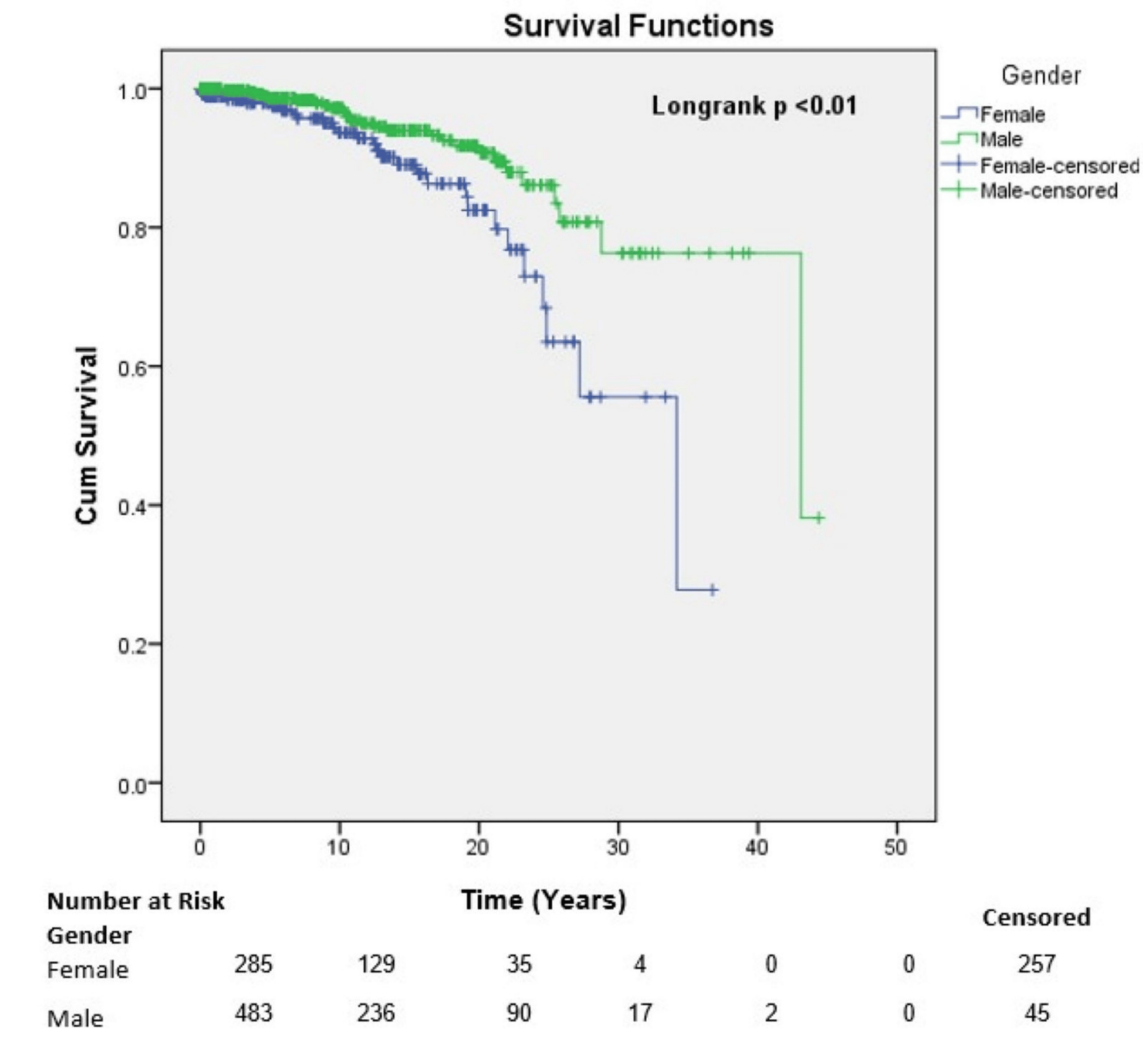
Survival of patients at risk by gender distribution.

The adjusted Kaplan-Meier survival curves further substantiate these findings (Figure 1).

The probability of survival for males and females at 25 years was approximately 86.0% and 63.0%, respectively. When comparing males to females, the probability of survival at 5 and 20 years was 99.0% versus 98.0%, and 92.0% versus 83.0%, respectively.

Association of valve replacement, type of valves, and NYHA class with survival outcomes

**Figure 2:**
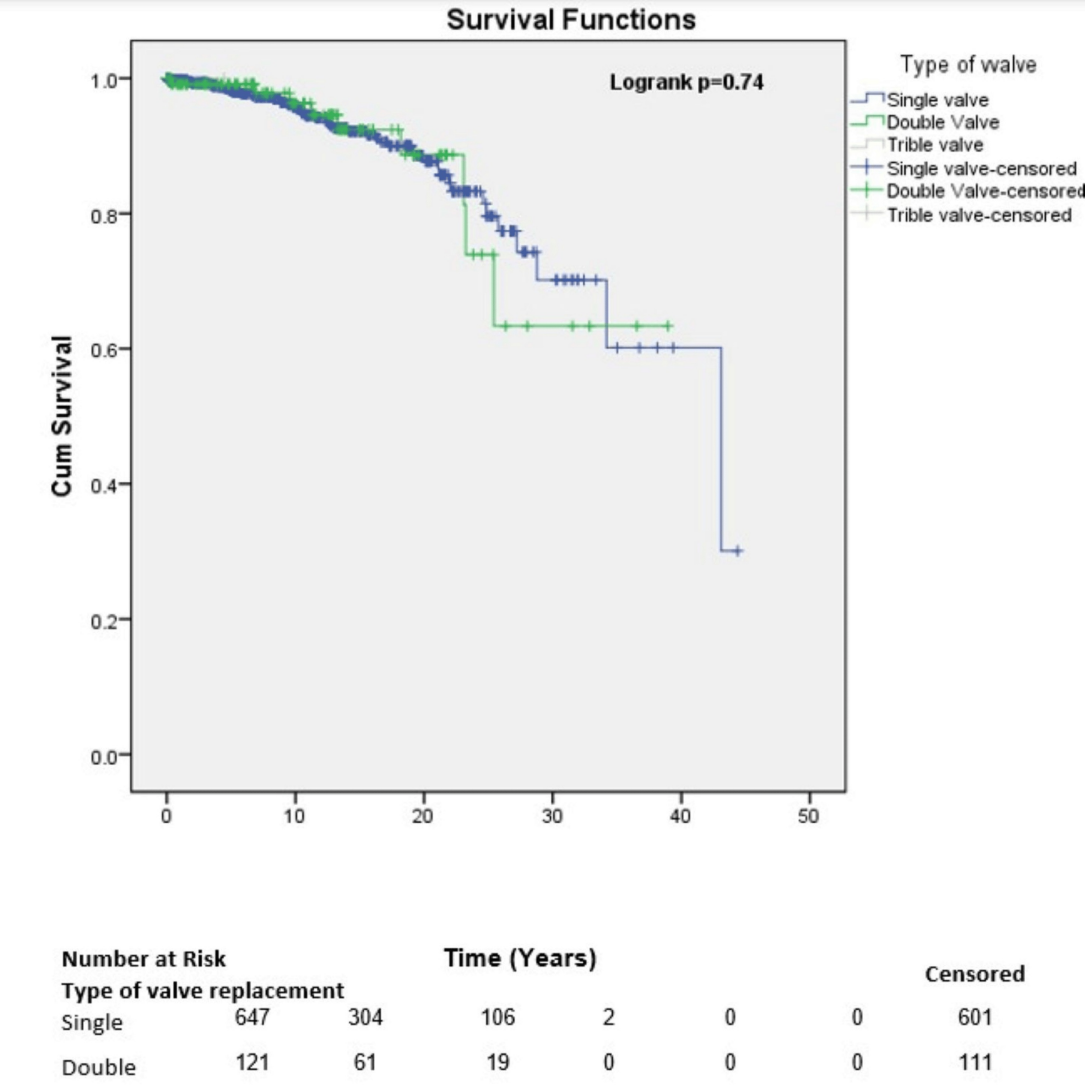
Survival of patients by type of valve used.

The estimates of mean survival time between patients with single valve replacement and double valve replacement were 35.6 years and 31.0 years, respectively (Figure 2).

**Figure 3:**
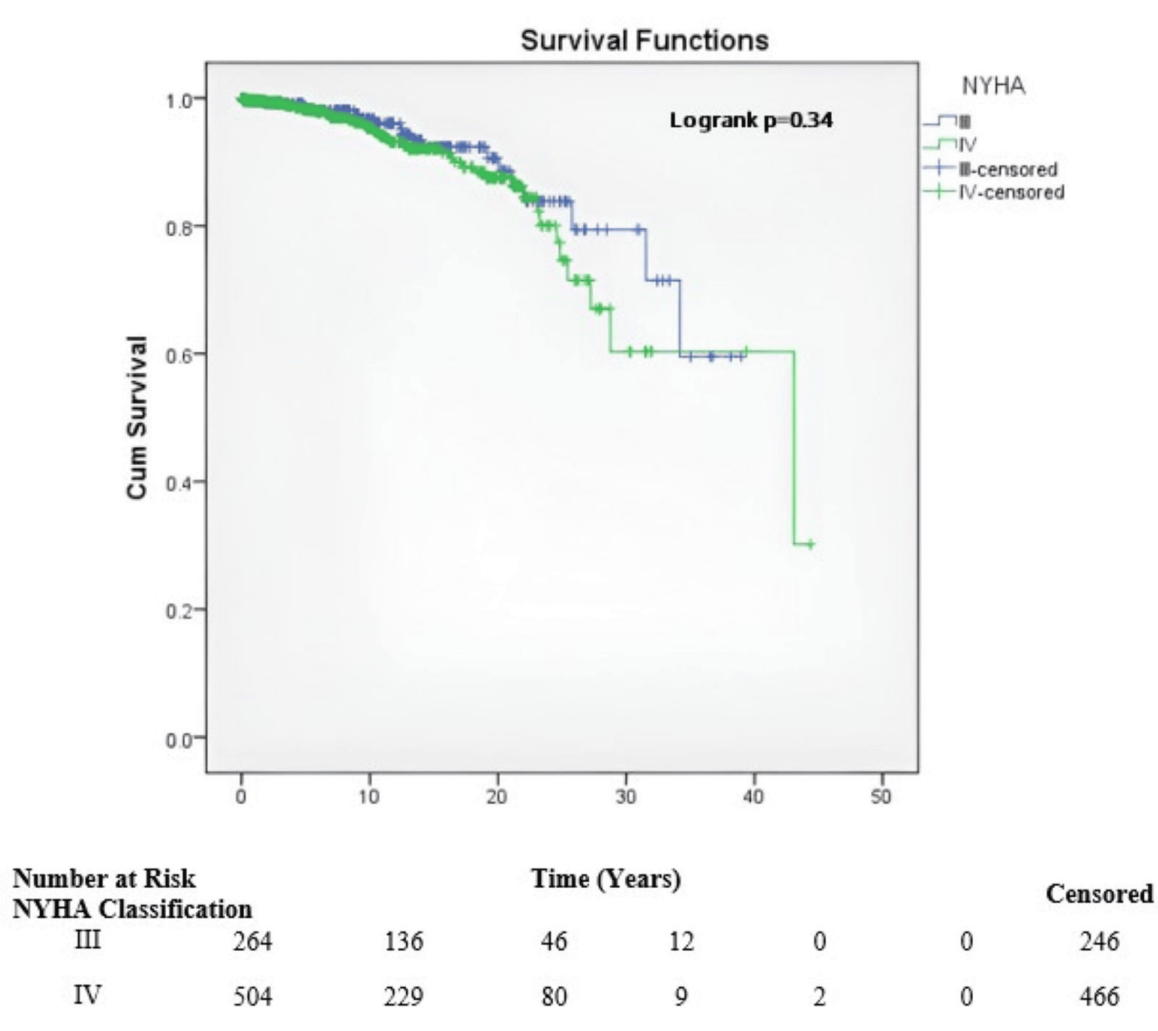
Survival of patients at risk by NYHA classification. NYHA: New York Heart Association.

After prosthetic valve replacement, a significant difference was observed between the mean estimated survival in patients with a monoleaflet valve and those with a bileaflet valve (36.6 years vs. 25.3 years, p=0.001) (Figure 3).

**Figure 4:**
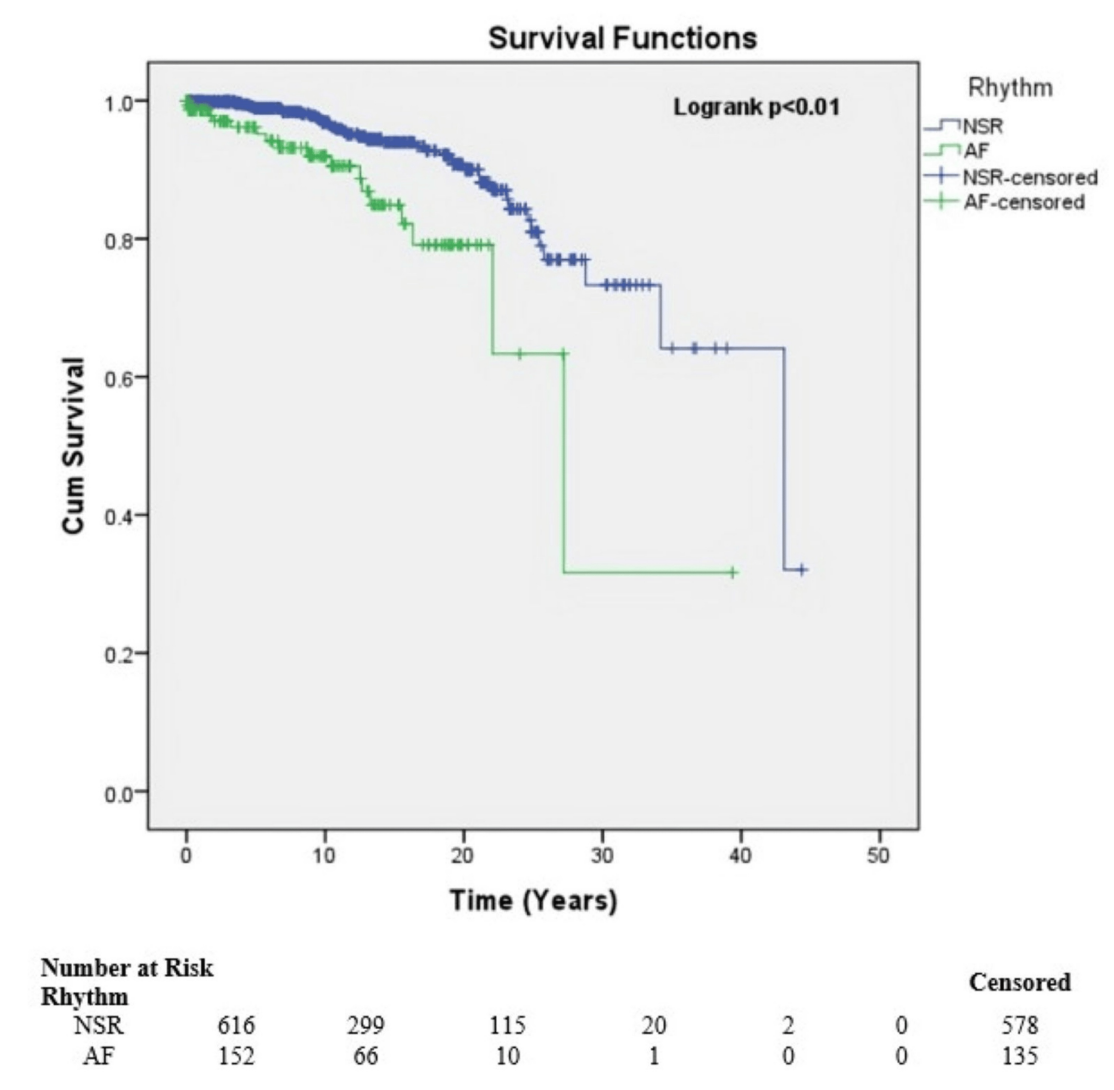
Survival of patients at risk by type of valve combination.

Of all the types of prosthetic valves used, monoleaflet valves such as Medtronic Hall (Antunes article) and Bjork-Shiley types were associated with better survival than other types (p=0.001) (Figure 4).

**Figure 5:**
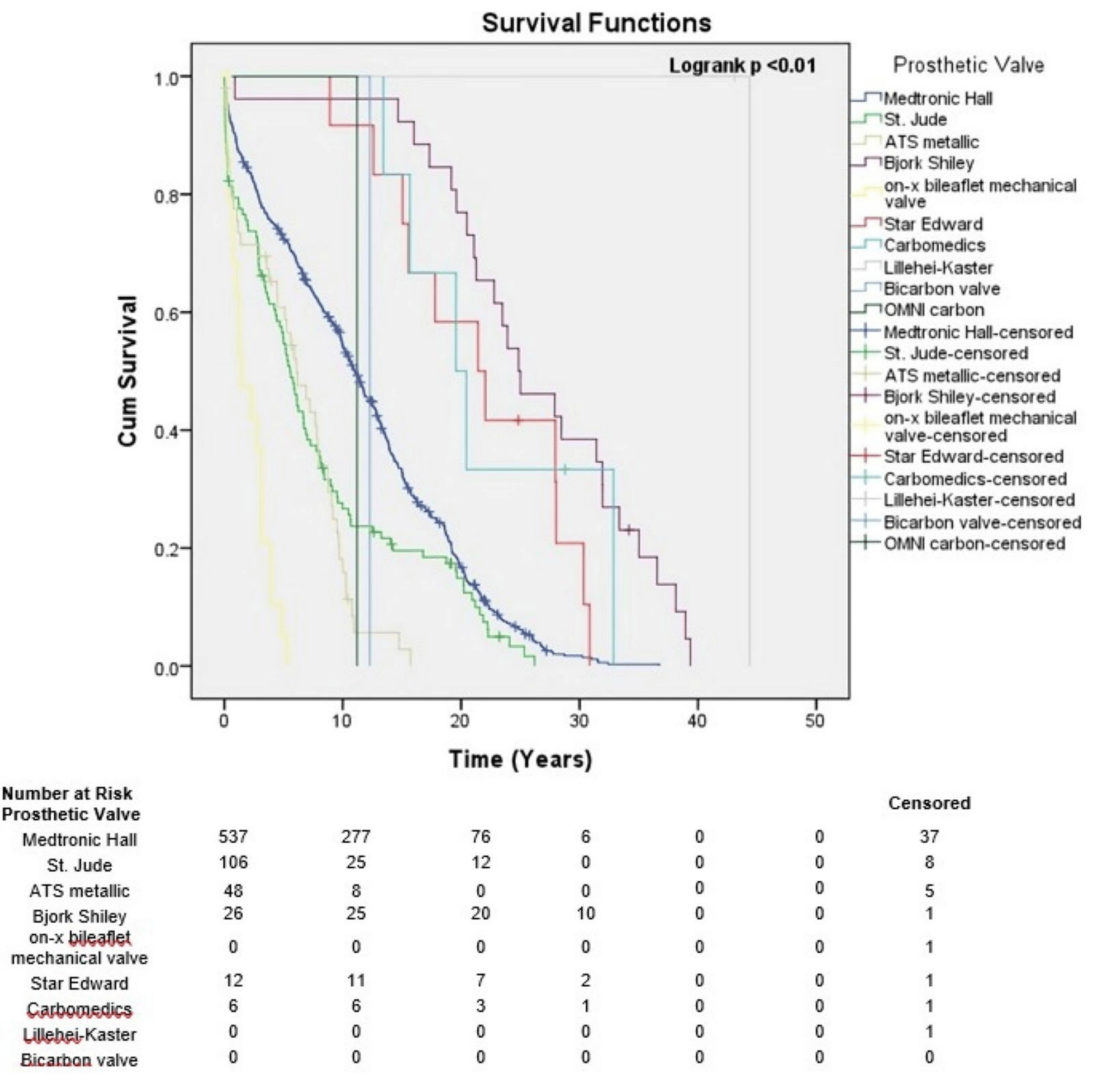
Survival of patients at risk by type of prosthetic valve.

The overall survival for patients classified as NYHA class III and IV was 33.44 years and 34.79 years, respectively (Figure 5).

Additionally, pannus excision was possible in redo surgery, particularly with the Medtronic Hall valve (p=0.09). The mean estimated survival of patients who underwent redo cardiac surgery was 33.0 years, compared with 34.9 years for those who did not. However, the difference in overall survival was not statistically significant.

Association between patient characteristics and overall survival

**Figure 6:**
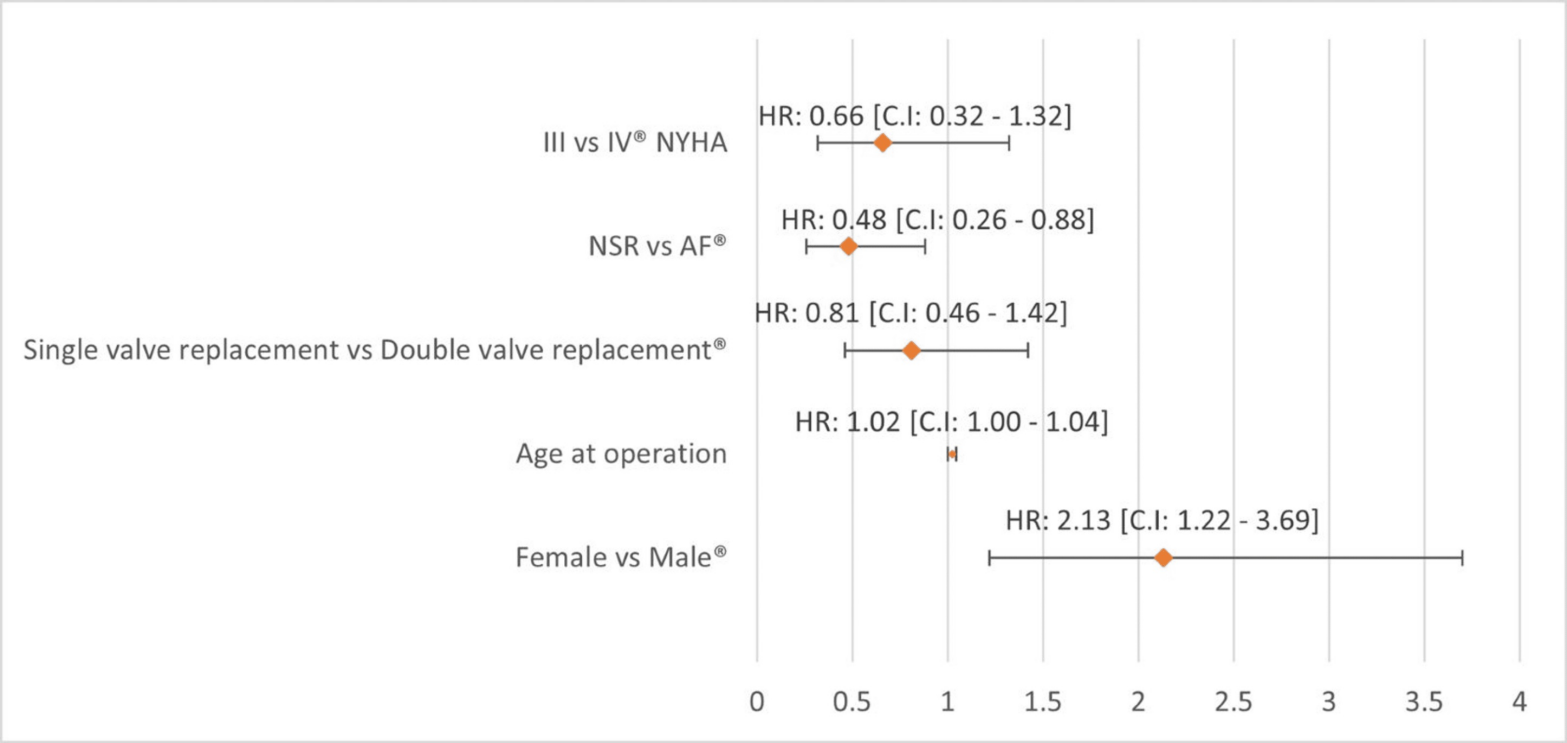
Forest plot of HRs (95% CIs) representing multivariate analysis of long-term survival.

HRs, stratified by characteristics such as age at valve replacement, sex, type of valve replacement, and NYHA class, were presented in Figure 6.

Women exhibited lower long-term survival than men, with a significant difference after prosthetic valve replacement (HR: 2.1; 95% CI: 1.2, 3.7). Interestingly, increasing age at valve replacement did not correlate with a difference in long-term survival within our cohort (HR: 1.02; 95% CI: 1.00, 1.04). Survival analysis revealed that patients undergoing single valve replacement did not significantly differ from those with double valve replacement (HR: 0.8; 95% CI: 0.46, 1.42). Notably, patients in normal sinus rhythm exhibited higher survival compared to those with atrial fibrillation, and this difference was statistically significant (HR: 0.48; 95% CI: 0.26, 0.88). Additionally, no significant difference in survival was observed between patients classified as NYHA functional class III and those in class IV (HR: 0.66; 95% CI: 0.32, 1.32). Further, despite 10 patients in NYHA class IV having severe cardiac cachexia, their long-term survival exceeded 10 years.

Time in TTR

**Figure 7:**
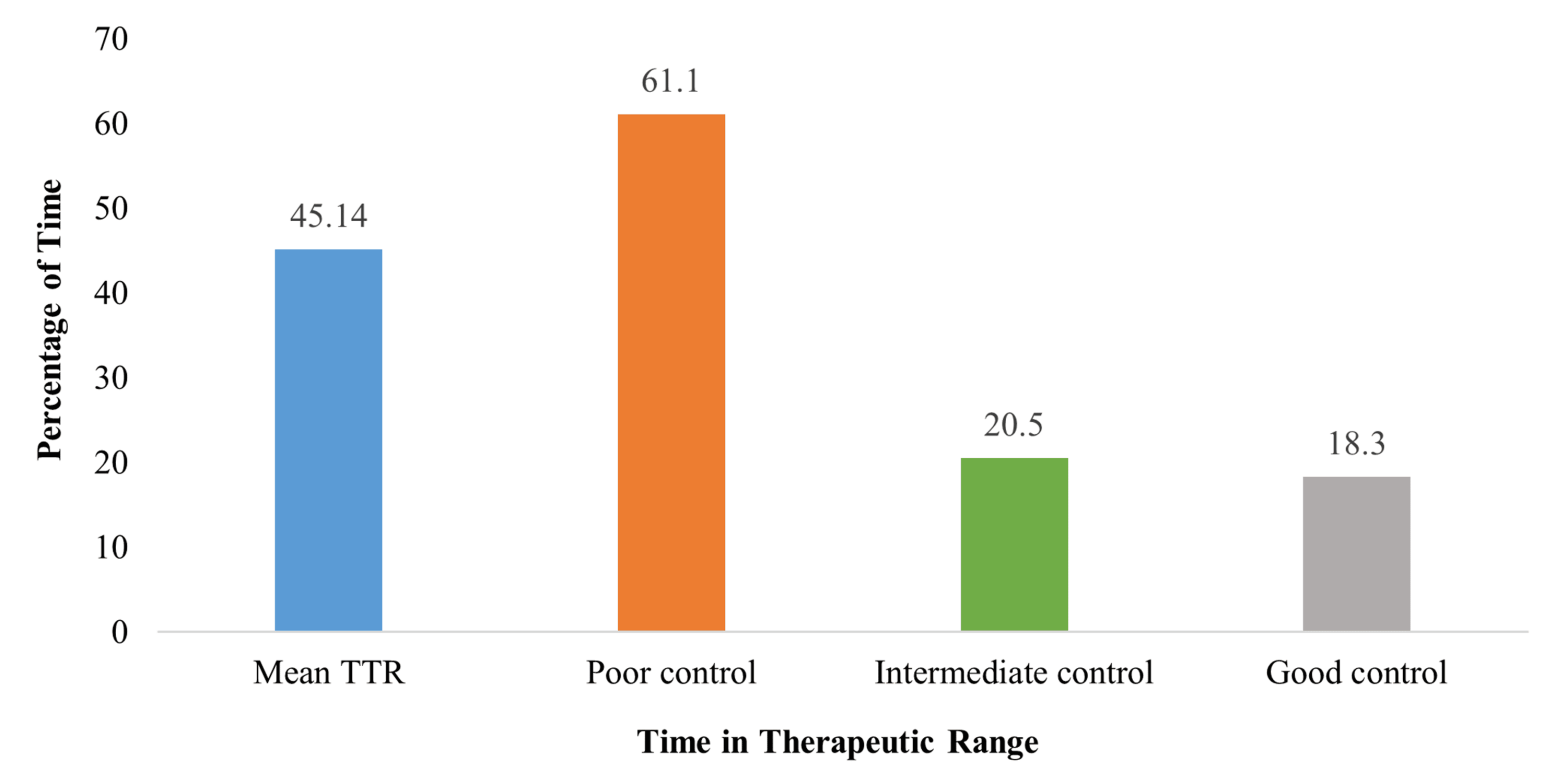
Relative distribution of TTR control groups. Poor control: TTR ≤ 50%; Intermediate control: TTR 50.1%-70.0%; Good control: TTR > 70%. TTR: Time to therapeutic range.

In this study, long-term INR control was assessed using the percentage of TTR, categorized into the following cut-off values: good TTR (>70%), intermediate TTR (50.1%-70%), and poor TTR (≤50%). The mean TTR score of our cohort was 45.1%. Specifically, 467 (61.1%) patients exhibited poor TTR control (≤50%), and 157 (20.5%) had intermediate TTR control. Notably, during the available study period, 140 (18.3%) patients achieved good TTR control (Figure 7).

## Discussion

This study might be the first of its kind to provide a detailed contemporary benchmark of long-term survival after mechanical valve replacement among patients in India. The observations reported from this study after a prolonged follow-up period can serve as a reference point for future studies and clinical trials, aiding healthcare providers in assessing and improving patient outcomes. Furthermore, this study was instrumental in identifying parameters that could be associated with better clinical and survival outcomes, further aiding in risk stratification and the optimization of management plans.

In India, the challenge lies in providing access to essential health services at an affordable cost. Among cardiovascular diseases (CVDs), heart valve disorders significantly impact healthcare utilization and costs [12-15]. In such scenarios, younger patients often require interventions that offer both durability and affordability. Bioprosthetic valves (BPLs), with their lower durability and increased risk of reoperations, impose a financial burden on patients. Hence, mechanical valves are often favored in younger age groups [12-14]. Although mechanical valves necessitate lifelong anticoagulation therapy, which may contribute to increased costs, this expense is relatively lower than that incurred from redo operations with BPLs. The possibility of redo operations is not acceptable to some patients due to social and financial constraints. Therefore, mechanical valves should be preferred in younger patients, as the benefits they offer outweigh the associated risks [16-18].

Gender disparity was identified as one of the factors affecting survival outcomes. The probability of survival for males and females at 5, 20, and 25 years was 99.0% vs. 98.0%, 92.0% vs. 83.0%, and 86.0% vs. 63.0%, respectively. It is quite apparent from this data that females have lower long-term survival rates than males, which might be attributed to social factors favoring better long-term medical care for males.

Female sex, advanced age, higher NYHA class, and poor LV function were associated with poor long-term outcomes in patients with double valve replacement [19]. In this study, the 1-year cumulative incidence of MACCE events in patients managed with acenocoumarol was 11.17 events per 100 person-years. However, a trend toward lower risk of MACCE events at 5 years was observed, likely due to vitamin K antagonists (VKAs) eliciting broader interference with the coagulation cascade. In the first year post-surgery, females experienced MACCE at a rate of 11.1 person-years, while males had none. Similar observations were reported in studies by Chaker Z et al., where females demonstrated worse in‐hospital mortality following AVR compared with men [18]. This was consistent with another study by Onorati F et al., where female sex was identified as an independent risk factor for mortality after aortic valve replacement [19]. However, it is sobering to note that despite a tendency toward a higher incidence of MACCE in women at 1-year post-valve replacement, this study suggests a trend toward lower MACCE complications after a mean follow-up of 5 years.

A study by Giustino G et al. concluded that advanced NYHA functional class was strongly associated with a higher risk for adverse events and poor health status outcomes [20]. This agrees with the present study, where among patients with NYHA functional class III and IV symptoms, the incidence of MACCE was higher in patients with NYHA class IV (8.38 per 100 person-years) compared with those with class III symptoms at 1 year.

Post-MHV surgery, it's advised to maintain an initial INR target range of 2.5 to 3.0 along with low-dose aspirin for the first 3 months. Later, the INR target can be lowered to 1.5 to 2.0 while continuing low-dose aspirin, although patients at high risk for thrombosis should stick to the higher INR target. Current guidelines suggest an INR range of 2.5 to 3.5 for mitral mechanical valves (MVs) [20-23]. In this study, despite the dose variations of acenocoumarol, mean INR levels generally remained within the therapeutic range of 2.0 to 3.5, ensuring appropriate anticoagulation management throughout the study period.

A study by Song HK et al. identified the removal of pannus as a safe and effective procedure, demonstrating satisfactory early clinical outcomes in cases of pannus overgrowth in mechanical aortic valves [23]. Consistent with this finding, the current study also revealed improved survival outcomes for those who underwent pannus excision in both aortic and mitral Medtronic Hall valves while preserving the original valves.

In this study, the type of valve replacement was identified as a factor affecting survival outcomes. A prospective study of 493 patients with mechanical heart valves (MHV) demonstrated that double valve replacement (DVR) was associated with better late survival and comparable in-hospital mortality when compared with isolated or single mitral valve replacement [24-28]. This contrasts with the present study, where patients who received single valve replacement (35.6 years) tended to have slightly better survival compared to those who underwent DVR (31.0 years).

The selection of optimal prosthetic valves for patients with VHD has been an unresolved dilemma for cardiac surgeons over the past few decades. There is a need to carefully balance the potential risks associated with BPLs, such as structural deterioration, infective endocarditis, paravalvular leak, and thrombosis or pannus formation in mechanical aortic valves against the survival benefits these valves may offer [15, 29, 30].

Research gaps

Women with RHD often neglect their health, leading to delayed diagnosis and treatment. There is a lack of research focusing on gender-specific challenges and strategies to improve early detection and management in women. Socioeconomic differences significantly affect access to timely treatment for RHD. Studies are needed to explore interventions that can mitigate these disparities and improve healthcare accessibility for disadvantaged populations. In cases where diagnosis is delayed, the disease often progresses to a stage where surgical repair is no longer feasible. There is a gap in understanding how to identify and manage such late-stage cases effectively to improve patient outcomes. Additionally, anticoagulation therapy, a crucial aspect of RHD management, is often overlooked in India. Further research is required to identify barriers to its adoption and to develop strategies for increasing awareness and adherence to this treatment modality.

Strengths

This study comprises longitudinal data spanning over a period of 40 years, involving 768 valve replacements performed by a single surgeon. Such an extended duration of follow-up is rare and offers valuable insights into the durability and long-term outcomes of mechanical valve replacements. This study helps treatment decisions and long-term management according to a population-specific need in Indian patients, who often undergo valve replacements at a younger age compared to their Western counterparts. We have evaluated not only survival outcomes but also postoperative complications and reoperation rates, thus providing a comprehensive picture of the long-term efficacy and safety of mechanical valve replacement in this population.

Limitations

Although the initial dataset comprised over 1,000 patients, a comparatively smaller sample of 768 patients was available for subgroup analysis. As this was a retrospective study, it is subject to selection bias and incomplete data. Moreover, since the study was non-randomized, it limits the ability to draw definitive conclusions about the comparative effectiveness of different mechanical valve replacement strategies or anticoagulation protocols.

However, it is important to note that despite these limitations, this study contributes valuable insights into the long-term outcomes and management challenges associated with mechanical valve replacement in Indian patients with VHD.

## Conclusions

This study contributes valuable insights into the long-term outcomes and management strategies for patients undergoing mechanical cardiac valve replacement. It demonstrated a mean survival rate of 35.2%, with men exhibiting higher survival rates compared to women. Over the prolonged follow-up period, survival outcomes were influenced by factors such as age, sex, type of valve replacement, and NYHA class, with certain subgroups showing better survival rates. Male gender, younger age, single-valve replacement, use of Bjork-Shiley type mechanical valves, normal sinus rhythm, and absence of advanced NYHA class were associated with more favorable survival outcomes. The study also underscores the need for further research and optimization of postoperative anticoagulation protocols to improve patient outcomes, particularly in the context of RHD, where significant evidence gaps and unmet needs persist, especially in regions like India.
